# Rebuttal From Dr Nadig et al

**DOI:** 10.1016/j.chpulm.2024.100068

**Published:** 2024-06-11

**Authors:** Tejaswi R. Nadig, Robert Smyth, Brett C. Bade

**Affiliations:** a Section of Interventional Pulmonology, Department of Medicine, Memorial Sloan Kettering Cancer Center, New York City, NY; b Section of Pulmonary, Critical Care, and Occupational Medicine, Department of Internal Medicine, University of Iowa, Iowa City, IA; c Section of Pulmonary, Critical Care, and Sleep Medicine, Department of Medicine, Northwell Health, New York City, NY

We appreciate the opportunity to respond to Kim et al,^1^ supporting the use of liquid biomarkers for risk stratification of pulmonary nodules. To start, Kim et al are to be commended for their thoughtful argument. Both teams agree that liquid biomarkers have the potential to improve clinical decision-making. However, both papers also highlight current limitations in biomarker use. Notably, candidate biomarkers are in the preclinical or clinical validation stages of development (Fig 1). Indeed, these limitations underscore our view that there is currently insufficient evidence to support the routine use of biomarkers for nodule management in clinical practice.^1^^,^^2^ It is noteworthy that both groups cite the same literature, but differ on the interpretation of the findings. Therefore, we wish to highlight two points in our response.

**Figure 1:**
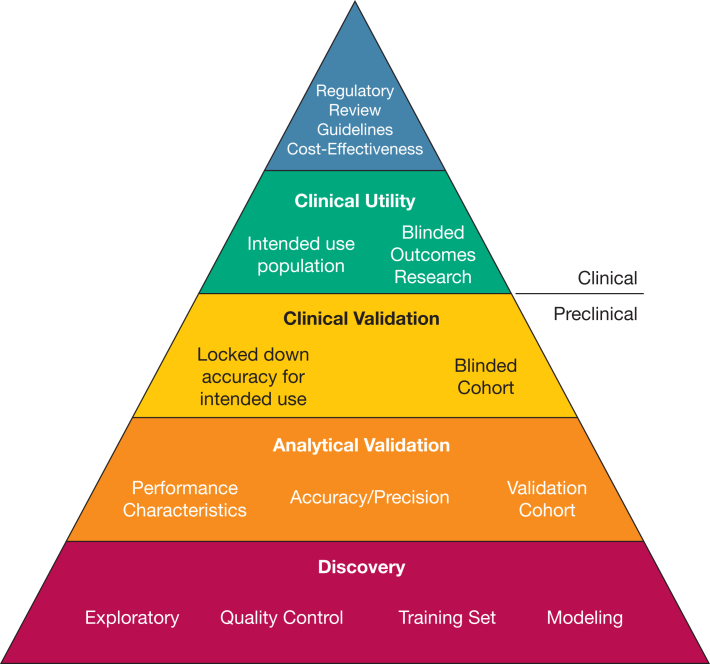
Stages of biomarker development. Reprinted with permission from Sears et al^3^

First, signals of potential clinical benefit should not be equated with readiness for widespread clinical use. Optimism from early data must be balanced against possible harm from premature implementation. The example of hydroxychloroquine as a therapy for COVID-19 comes to mind.^4^ In the setting of an urgent need and without viable alternatives or guidelines, hydroxychloroquine was widely used. With subsequent studies showing no clinical benefit from hydroxychloroquine in COVID-19, there was clearly potential for unintended risk without benefit. Similarly, if pulmonologists endorse widespread use of biomarkers for lung nodule assessment without sufficient evaluation, the risk of worsened outcomes (eg, delayed cancer diagnoses, invasive procedures for benign conditions) may also be increased. We again highlight the work by Tanner et al,^5^ suggesting that without proper education, biomarker implementation may not have the intended effect.

Second, in our zeal to reduce the number of invasive procedures (and associated complications) during lung nodule evaluation, we should not circumvent established processes for biomarker decision-making. As outlined by our colleagues, a clinical utility study^6^ and a clinical validation study^7^ are currently underway. Because many liquid biomarkers have undergone testing, yet few have reached clinical utility testing, the distance between may help and does help can be wide. Put another way, we cannot tailor the definition of readiness. Policy statements are painstakingly developed using a rigorous process to ensure recommendations are valid and reliable. This approach is essential in promoting transparency, consistency, and (ultimately) widespread implementation of the best test.^8^

Therefore, we reiterate that the criterion standard benchmarks established by the lung cancer community (and outlined in the American Thoracic Society policy statement) must be met.^8^, ^9^, ^10^, ^11^ Pulmonologists should insist on serum biomarkers completing their current clinical utility testing and attaining the standards required for a clinically effective biomarker: (1) improving clinical decision-making, (2) reducing unnecessary procedures, and (3) lowering lung cancer mortality. Only then should liquid biomarkers be incorporated into guideline-based clinical care (prime time).

In summary, we agree with Kim et al that preliminary data are exciting, and we are closer than ever to the inclusion of liquid biomarkers in lung nodule assessment. However, for the safety of the patients, it is incumbent on pulmonologists to await the results of ongoing trials to ensure these novel diagnostic approaches are indeed ready for prime time.

## Funding/Support

This study did not receive financial support.

## Financial/Nonfinancial Disclosures

The authors have reported to *CHEST Pulmonary* the following: B. C. B. reports relationships with the American Cancer Socity, the VA Central Office, Delfi Diagnostics, Biodesix, Inc, and Nucleix Ltd that includes site principal investigator for clinical trial. None declared (T. R. N. and R. S.).
